# Supplementary material for: Resolving the full spectrum of human genome variation using Linked-Reads
Source: Genome Res. 2019 Apr;29(4):635–45. doi: 10.1101/gr.234443.118 (PMC6442396; doi:10.1101/gr.234443.118)
Supplement: Supplemental Material [file supp_gr.234443.118_Supplemental_longranger-2.2.zip › longranger-2.2.tar.bz2!/lr2.2-public/lib/stan/cmdstan/stan/doc/api/html/classstan_1_1mcmc_1_1windowed__adaptation.html]

Stan: stan::mcmc::windowed\_adaptation Class Reference


|  |  |
| --- | --- |
|  | Stan  2.5.0  probability, sampling & optimization |


- Main Page
- Related Pages
- Namespaces
- Classes
- Files

- Class List
- Class Index
- Class Hierarchy
- Class Members

All Classes Namespaces Files Functions Variables Typedefs Enumerations Enumerator Friends Macros Pages

- stan
- mcmc
- windowed\_adaptation

Public Member Functions |
Protected Attributes |
List of all members

stan::mcmc::windowed\_adaptation Class Reference

`#include <windowed_adaptation.hpp>`

Inheritance diagram for stan::mcmc::windowed\_adaptation:

|  |  |
| --- | --- |
| Public Member Functions | |
|  | windowed\_adaptation (std::string name) |
|  | |
| void | restart () |
|  | |
| void | set\_window\_params (unsigned int num\_warmup, unsigned int init\_buffer, unsigned int term\_buffer, unsigned int base\_window, std::ostream \*e=0) |
|  | |
| bool | adaptation\_window () |
|  | |
| bool | end\_adaptation\_window () |
|  | |
| void | compute\_next\_window () |
|  | |

|  |  |
| --- | --- |
| Protected Attributes | |
| std::string | estimator\_name\_ |
|  | |
| unsigned int | num\_warmup\_ |
|  | |
| unsigned int | adapt\_init\_buffer\_ |
|  | |
| unsigned int | adapt\_term\_buffer\_ |
|  | |
| unsigned int | adapt\_base\_window\_ |
|  | |
| unsigned int | adapt\_window\_counter\_ |
|  | |
| unsigned int | adapt\_next\_window\_ |
|  | |
| unsigned int | adapt\_window\_size\_ |
|  | |

## Detailed Description

Definition at line 13 of file windowed\_adaptation.hpp.

## Constructor & Destructor Documentation

|  |  |  |  |  |  |
| --- | --- | --- | --- | --- | --- |
| stan::mcmc::windowed\_adaptation::windowed\_adaptation | ( | std::string | *name* | ) |  |

inline

Definition at line 17 of file windowed\_adaptation.hpp.

## Member Function Documentation

|  |  |  |  |  |
| --- | --- | --- | --- | --- |
| bool stan::mcmc::windowed\_adaptation::adaptation\_window | ( |  | ) |  |

inline

Definition at line 77 of file windowed\_adaptation.hpp.

|  |  |  |  |  |
| --- | --- | --- | --- | --- |
| void stan::mcmc::windowed\_adaptation::compute\_next\_window | ( |  | ) |  |

inline

Definition at line 88 of file windowed\_adaptation.hpp.

|  |  |  |  |  |
| --- | --- | --- | --- | --- |
| bool stan::mcmc::windowed\_adaptation::end\_adaptation\_window | ( |  | ) |  |

inline

Definition at line 83 of file windowed\_adaptation.hpp.

|  |  |  |  |  |
| --- | --- | --- | --- | --- |
| void stan::mcmc::windowed\_adaptation::restart | ( |  | ) |  |

inlinevirtual

Reimplemented from stan::mcmc::base\_adaptation.

Definition at line 26 of file windowed\_adaptation.hpp.

|  |  |  |  |
| --- | --- | --- | --- |
| void stan::mcmc::windowed\_adaptation::set\_window\_params | ( | unsigned int | *num\_warmup*, |
|  |  | unsigned int | *init\_buffer*, |
|  |  | unsigned int | *term\_buffer*, |
|  |  | unsigned int | *base\_window*, |
|  |  | std::ostream \* | *e* = `0` |
|  | ) |  |  |

inline

Definition at line 32 of file windowed\_adaptation.hpp.

## Member Data Documentation

unsigned int stan::mcmc::windowed\_adaptation::adapt\_base\_window\_

protected

Definition at line 115 of file windowed\_adaptation.hpp.

unsigned int stan::mcmc::windowed\_adaptation::adapt\_init\_buffer\_

protected

Definition at line 113 of file windowed\_adaptation.hpp.

unsigned int stan::mcmc::windowed\_adaptation::adapt\_next\_window\_

protected

Definition at line 118 of file windowed\_adaptation.hpp.

unsigned int stan::mcmc::windowed\_adaptation::adapt\_term\_buffer\_

protected

Definition at line 114 of file windowed\_adaptation.hpp.

unsigned int stan::mcmc::windowed\_adaptation::adapt\_window\_counter\_

protected

Definition at line 117 of file windowed\_adaptation.hpp.

unsigned int stan::mcmc::windowed\_adaptation::adapt\_window\_size\_

protected

Definition at line 119 of file windowed\_adaptation.hpp.

std::string stan::mcmc::windowed\_adaptation::estimator\_name\_

protected

Definition at line 110 of file windowed\_adaptation.hpp.

unsigned int stan::mcmc::windowed\_adaptation::num\_warmup\_

protected

Definition at line 112 of file windowed\_adaptation.hpp.

---

The documentation for this class was generated from the following file:

- src/stan/mcmc/windowed\_adaptation.hpp


---

[ Stan Home Page ]

*© 2011–2014,
Stan Development Team.*
